# Lidocaine enhances antitumor effects of sorafenib and GW5074 in colorectal cancer cells

**DOI:** 10.1080/14756366.2026.2678755

**Published:** 2026-06-08

**Authors:** Tzu-Chiao Lin, Zih-Syuan Wu, Shih-Ming Huang, Che-Wei Liu, Je-Ming Hu, Chia-Cheng Wen

**Affiliations:** a Division of Colon and Rectal Surgery, Department of Surgery, School of Medicine, College of Medicine, Tri-Service General Hospital, National Defense Medical University, Taipei, Taiwan Republic of China; b Graduate Institute of Biochemistry, College of Biomedical Sciences, National Defense Medical University, Taipei City, Taiwan, Republic of China

**Keywords:** Lidocaine, sorafenib, GW5074, colorectal cancer, combination index

## Abstract

Targeted therapies have broadened the treatment landscape for colorectal cancer (CRC), highlighting the need for rational combination strategies. Although sorafenib and GW5074 show therapeutic promise and lidocaine may sensitise cancer cells to treatment, the mechanisms underlying these combinations remain poorly understood. Here, we systematically evaluated the effects of combining lidocaine with sorafenib and/or GW5074 in CRC cell lines. Combination index analysis showed that sorafenib increased responsiveness to GW5074 by 19.5- and 1.7 × 10^7^-fold in HT29 and SW480 cells, respectively, while lidocaine enhanced this response by 45- and 3 × 10^7^-fold. Ropivacaine, bupivacaine, and levobupivacaine also showed synergistic interactions with GW5074 by 14- and 1.03 × 10^7^-fold, 15.1- and 6.5 × 10^6^-fold, and 33.6- and 3.8 × 10^7^-fold in HT29 and SW480 cells, respectively. Lidocaine and ropivacaine in combination with sorafenib exhibited an antagonistic effect in HT29 cells, others were synergistic effects. These combinations modulated cell-cycle progression, activated cell death pathways, and amplified cellular stress responses in a context-dependent manner.

## Introduction

Colorectal cancer (CRC) is the third most commonly diagnosed cancer worldwide and a leading cause of cancer-related mortality, highlighting the need for improved diagnostic and therapeutic strategies^1^. Although surgery, chemotherapy, and radiotherapy remain central to CRC management, treatment is often limited by resistance, toxicity, and suboptimal long-term efficacy^2^. Despite recent advances, metastatic CRC (mCRC) continues to have a poor prognosis, with an approximately 14% 5-year survival rate^3^. Targeted therapies have expanded treatment options, but their clinical effectiveness remains limited, prompting interest in rational combination strategies.

Sorafenib is an oral multikinase inhibitor that targets multiple signalling pathways, including the Raf–MEK–ERK cascade, vascular endothelial growth factor receptor, and platelet-derived growth factor receptor (PDGFR)^4^. It is FDA approved for advanced hepatocellular carcinoma, progressive renal cell carcinoma, and radioactive iodine-refractory differentiated thyroid carcinoma. Preclinical and clinical studies have shown that sorafenib exerts antitumor effects in CRC models, either alone or combined with agents such as erlotinib, cetuximab, or bevacizumab^5^. In mCRC, sorafenib plus capecitabine has improved progression-free and overall survival^6^. However, acquired resistance remains a major limitation, highlighting the need for combination strategies that enhance therapeutic efficacy.

A key challenge in CRC treatment is frequent dysregulation of the Raf/MEK/ERK pathway, often driven by activating mutations in KRAS, NRAS, or BRAF^1^. Persistent pathway activation promotes tumour cell survival, proliferation, and resistance to standard therapies. GW5074, a selective C-RAF kinase inhibitor, has emerged as a potential anticancer agent. By inhibiting MAPK signalling, GW5074 may enhance the effects of chemotherapy and targeted therapy^7^. Previous studies showed that GW5074 increases sorafenib cytotoxicity by inducing mitochondrial dysfunction, suggesting a potential sensitising role^8^. In renal cell carcinoma models, sorafenib and GW5074 synergistically induced cancer cell death and inhibited tumour growth^9^.

Local anaesthetics, particularly lidocaine, have garnered attention as potential anticancer agents beyond their traditional role in pain management^10^^,^^11^. Evidence suggests that lidocaine inhibits tumour cell proliferation, migration, and invasion, modulates mitochondrial function, increases reactive oxygen species (ROS) production, and enhances tumour cell sensitivity to chemotherapy^10^^,^^12^^,^^13^. Lidocaine has also been shown to induce ferroptosis in ovarian and breast cancer cells by targeting the miR-382-5p/SLC7A11 axis^14^, increasing lipid peroxidation and reducing GPX4 activity. These findings suggest that lidocaine may have multifaceted anticancer effects.

Drug repurposing offers an efficient strategy for identifying anticancer agents while reducing development time and cost compared with *de novo* drug discovery^15^^,^^16^. In this context, local anaesthetics, including lidocaine, have been investigated as candidate anticancer agents^10^^,^^11^^,^^17^. Lidocaine enhances the cytotoxicity of several chemotherapeutic agents, including mitomycin C, pirarubicin, cisplatin, and 5-fluorouracil (5-FU)^10^^,^^18–20^. In this study, we investigated the effects of lidocaine combined with sorafenib, GW5074, or both in CRC cells. We aimed to determine whether these regimens enhance cytotoxicity, overcome resistance associated with targeted therapies, and modulate cell death pathways.

## Materials and methods

### Cell culture and reagents

HT29 and SW480 cell lines were obtained from the American Type Culture Collection (ATCC; Manassas, VA, USA). Cells were maintained in Roswell Park Memorial Institute (RPMI) 1640 medium, supplemented with 10% foetal bovine serum (FBS) and 1% penicillin/streptomycin solution (Invitrogen, Waltham, MA, USA). Lidocaine, sorafenib, GW5074, ropivacaine, bupivacaine, levobupivacaine, thiazolyl blue tetrazolium bromide (MTT), propidium iodide (PI), and acridine orange (Cat. No. A8097) were sourced from Sigma Aldrich (St. Louis, MO, USA).

#### Cell viability and combination index analysis

HT29 and SW480 cells (5 × 10³ cells/well) were seeded in 96-well plates and allowed to attach overnight. The following day, cells were treated with lidocaine, ropivacaine, bupivacaine, levobupivacaine, sorafenib, GW5074, or their combinations for 48 h. Cell viability was assessed using the MTT assay. MTT solution (0.5 mg/mL in PBS) was added to each well and incubated for 4 h at 37 °C. After supernatant removal, 100 μL DMSO was added to dissolve the formazan crystals. Absorbance was measured at 570 nm, with 650 nm as the reference wavelength, using a Varioskan LUX microplate reader (Thermo Fisher Scientific, Vantaa, Finland). Combination index (CI) values were calculated using CalcuSyn software (version 2; Biosoft, Cambridge, UK) to construct isobolograms. CI values <1 indicated synergism, and CI values >1 indicated antagonism^21^.

#### Cell-cycle profile analysis

HT29 and SW480 cells (5 × 10⁵ cells/well) were seeded in six-well plates and allowed to adhere overnight. The next day, cells were treated with lidocaine, sorafenib, GW5074, or their combinations for 48 h. Cells were then fixed in 70% ice-cold ethanol and stored at −20 °C overnight. After fixation, cells were centrifuged at 1000 rpm for 5 min, washed twice with ice-cold PBS containing 1% FBS, and incubated with PI staining solution containing 5 μg/mL PI, 0.5% Triton X-100, and 0.5 μg/mL RNase A in PBS for 30 min at 37 °C in the dark. Flow cytometry was performed using a BD FACSCalibur^™^, with 10 000 events acquired per sample. Data were analysed using CellQuest Pro software (version 5.1, BD Biosciences, Milpitas, CA, USA).

#### Colony formation assay

HT29 and SW480 cells (2 × 10³ cells/well) were seeded in six-well plates and allowed to adhere overnight. The next day, cells were treated with lidocaine, sorafenib, GW5074, or their combinations for 2 weeks. Colonies were fixed with methanol and stained with 0.005% crystal violet. Colonies larger than 0.05 mm were counted using ImageJ software (version 1.44a, NIH, Bethesda, MD, USA).

#### Apoptosis analysis

HT29 and SW480 cells (5 × 10⁵ cells/well) were seeded in six-well plates and allowed to adhere overnight. The next day, cells were treated with lidocaine, sorafenib, GW5074, or their combinations for 48 h. Cells were then rinsed twice with ice-cold PBS and resuspended in 200 μL binding buffer. Annexin V-PE (5 μL) and 7-AAD (10 μL, 5 μg/mL) were added, and samples were incubated for 15 min at 23 °C in the dark. Apoptotic cell populations were analysed using a BD FACSCalibur^™^ flow cytometer, and data were processed using CellQuest Pro software (version 5.1, BD Biosciences).

#### Acridine orange staining for autophagy

HT29 and SW480 cells (5 × 10⁵ cells/well) were seeded in six-well plates and cultured overnight. The next day, cells were treated with lidocaine, sorafenib, GW5074, or their combinations for 48 h. Cells were stained with acridine orange (AO; 1 µg/mL) for 30 min at 37 °C in the dark. After three PBS washes to remove unbound dye, cells were resuspended in fresh PBS and immediately analysed for fluorescence using a BD FACSCalibur^™^ flow cytometer. Data were acquired and analysed using CellQuest Pro software (version 5.1, BD Biosciences).

#### Reactive oxygen species (ROS) analysis

Intracellular ROS generation was assessed using DCFH-DA. HT29 and SW480 cells (5 × 10⁵ cells/well) were seeded in six-well plates and cultured overnight. The next day, cells were treated with lidocaine, sorafenib, GW5074, or the indicated combinations for 48 h. Cells were then incubated with DCFH-DA (20 μM) for 30 min at 37 °C in the dark, harvested, rinsed once with PBS to remove excess dye, and analysed for fluorescence intensity in the FL-1 channel using a BD FACSCalibur^™^ flow cytometer. Data were acquired and processed using CellQuest Pro software (version 5.1, BD Biosciences).

#### Lipid peroxidation analysis

Lipid peroxidation was assessed using C11-BODIPY (Thermo Fisher Scientific, D3861). HT29 and SW480 cells were treated with lidocaine, sorafenib, GW5074, or their combinations for 48 h and then incubated with 10 μM C11-BODIPY for 1 h at 37 °C. Cells were harvested, rinsed, and resuspended in PBS containing 1% BSA. Fluorescence intensity was measured using a BD FACSCalibur^™^ flow cytometer, and data were processed using CellQuest Pro software (version 5.1, BD Biosciences).

#### Hypoxic analysis

HT29 and SW480 cells (5 × 10⁵ cells/well) were seeded in six-well plates and cultured overnight. After treatment with lidocaine, sorafenib, GW5074, or their combinations for 48 h, cells were incubated with Hypoxia Red dye (ENZ-51042, Enzo Life Sciences) for 30 min at 37 °C. Cellular hypoxia levels were quantified using a BD FACSCalibur^™^ flow cytometer, and data were analysed using CellQuest Pro software (version 5.1, BD Biosciences).

#### Mitochondrial membrane potential analysis

HT29 and SW480 cells (5 × 10⁵ cells/well) were seeded in six-well plates and cultured overnight. After 48 h of treatment with lidocaine, sorafenib, GW5074, or their combinations, floating and adherent cells were collected, rinsed once with PBS, and incubated with JC-1 staining solution in 1× binding buffer for 30 min at 37 °C in the dark. Cells were then washed twice with PBS, resuspended in 500 µL binding buffer, and analysed using a BD FACSCalibur^™^ flow cytometer with CellQuest Pro software (version 5.1, BD Biosciences).

### Statistics

Bar graphs show individual data points representing biological replicates. For line graphs, the number of biological replicates is provided in the corresponding figure legends. Data are presented as mean ± standard error of the mean (SEM). Statistical analyses were performed using GraphPad Prism (version 9, GraphPad Software, San Diego, CA, USA). Depending on the experimental design, one-way ANOVA followed by Tukey’s *post hoc* test, two-way ANOVA followed by Dunnett’s multiple-comparisons test, or a two-tailed Student’s t-test was used. Error bars represent SEM. Statistical significance was defined as *p < 0.05, **p < 0.01, ***p < 0.001, and ****p < 0.0001.

## Results

### Evaluation of synergistic effects, cell-cycle modulation, and clonogenic suppression by sorafenib, GW5074, and lidocaine in CRC cells

**Figure 1.**
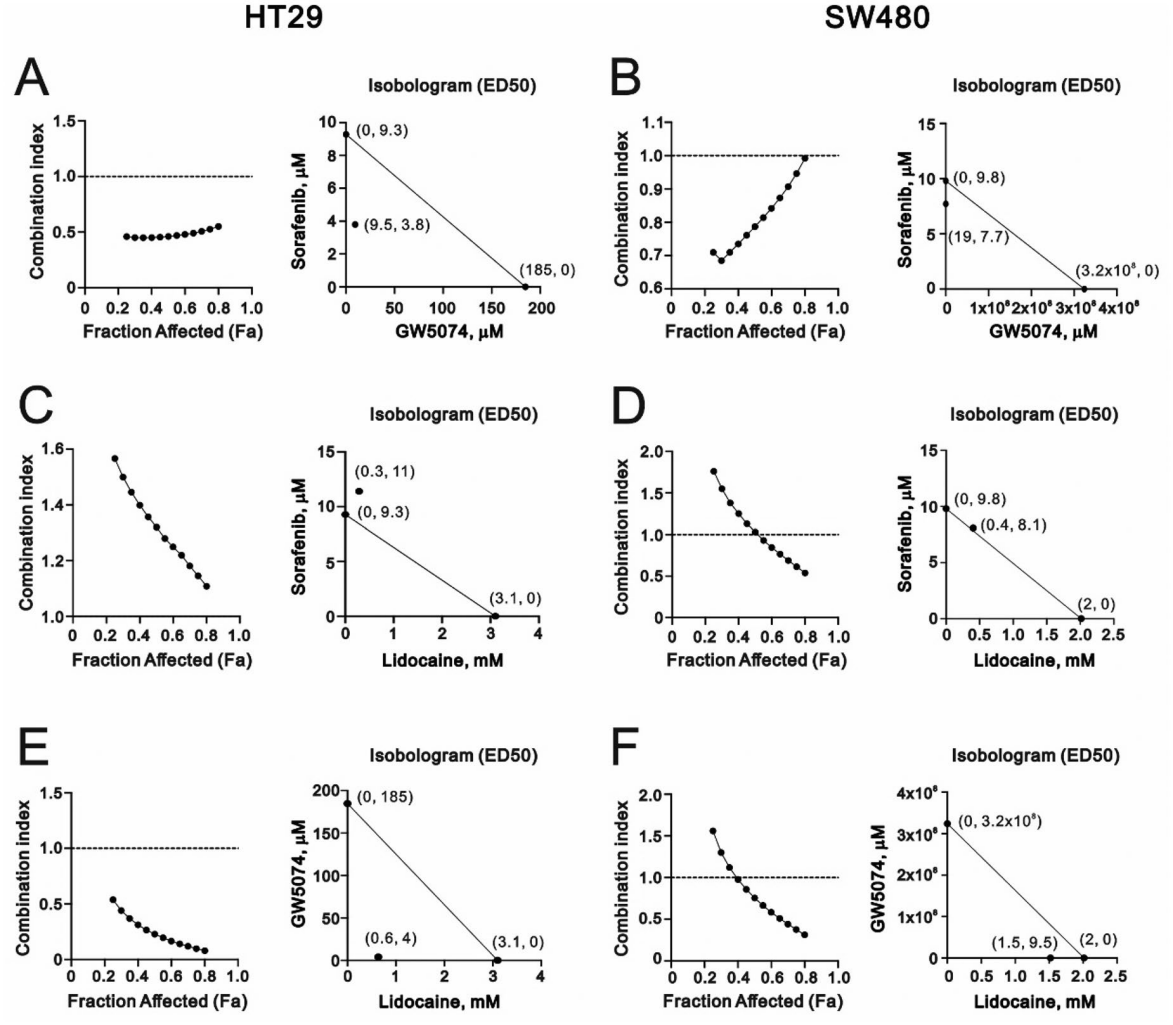
Assessment of drug interactions among sorafenib, GW5074, and lidocaine in CRC cells using combination index (CI) analysis. (A) HT29 and (B) SW480 cells were treated for 48 h with increasing concentrations of sorafenib (0, 0.625, 1.25, 2.5, 5, 10, 20, or 40 μM) in combination with GW5074 (0, 0.39, 0.78, 1.56, 3.12, 6.25, 12.5, 25, 50, or 100 μM). (C) HT29 and (D) SW480 cells were treated for 48 h with increasing concentrations of lidocaine (0, 0.125, 0.25, 0.5, 1, 2, 4, or 8 mM) in combination with sorafenib (0, 0.625, 1.25, 2.5, 5, 10, 20, 40, 80, or 160 μM). (E) HT29 and (F) SW480 cells were treated for 48 h with increasing concentrations of lidocaine (0, 0.125, 0.25, 0.5, 1, 2, 4, or 8 mM) in combination with GW5074 (0, 0.39, 0.78, 1.56, 3.12, 6.25, 12.5, 25, 50, or 100 μM). Cell viability was assessed using the MTT assay. ED50 isobolograms and CI plots were generated using CalcuSyn software.

We used two colon cancer cell lines: HT29 and SW480. HT29 is derived from a more advanced Dukes stage C adenocarcinoma (T2–3 N1 M0 grade), whereas SW480 is derived from a primary Dukes stage B adenocarcinoma (T2–3 N0 M0 grade)^22^. To assess potential synergistic interactions among sorafenib, GW5074, and lidocaine in CRC cells, we first calculated the CI. Sorafenib and GW5074 showed synergistic effects in both HT29 and SW480 cells, with CI values below 1 (Figure 1(A,B)). In HT29 cells, lidocaine plus sorafenib showed a CI above 1, indicating antagonism (Figure 1(C)), whereas lidocaine plus GW5074 showed synergistic effects (Figure 1(E)). In SW480 cells, lidocaine combined with either sorafenib (Figure 1(D)) or GW5074 (Figure 1(F)) showed synergistic interactions. Sorafenib increased GW5074 responsiveness by 19.5-fold in HT29 cells and 1.7 × 10^7^-fold in SW480 cells, whereas lidocaine enhanced GW5074 responsiveness by 45-fold and 3 × 10^7^-fold, respectively. Thus, the GW5074–lidocaine combination was more effective than the sorafenib–GW5074 combination in both cell lines.

**Figure 2.**
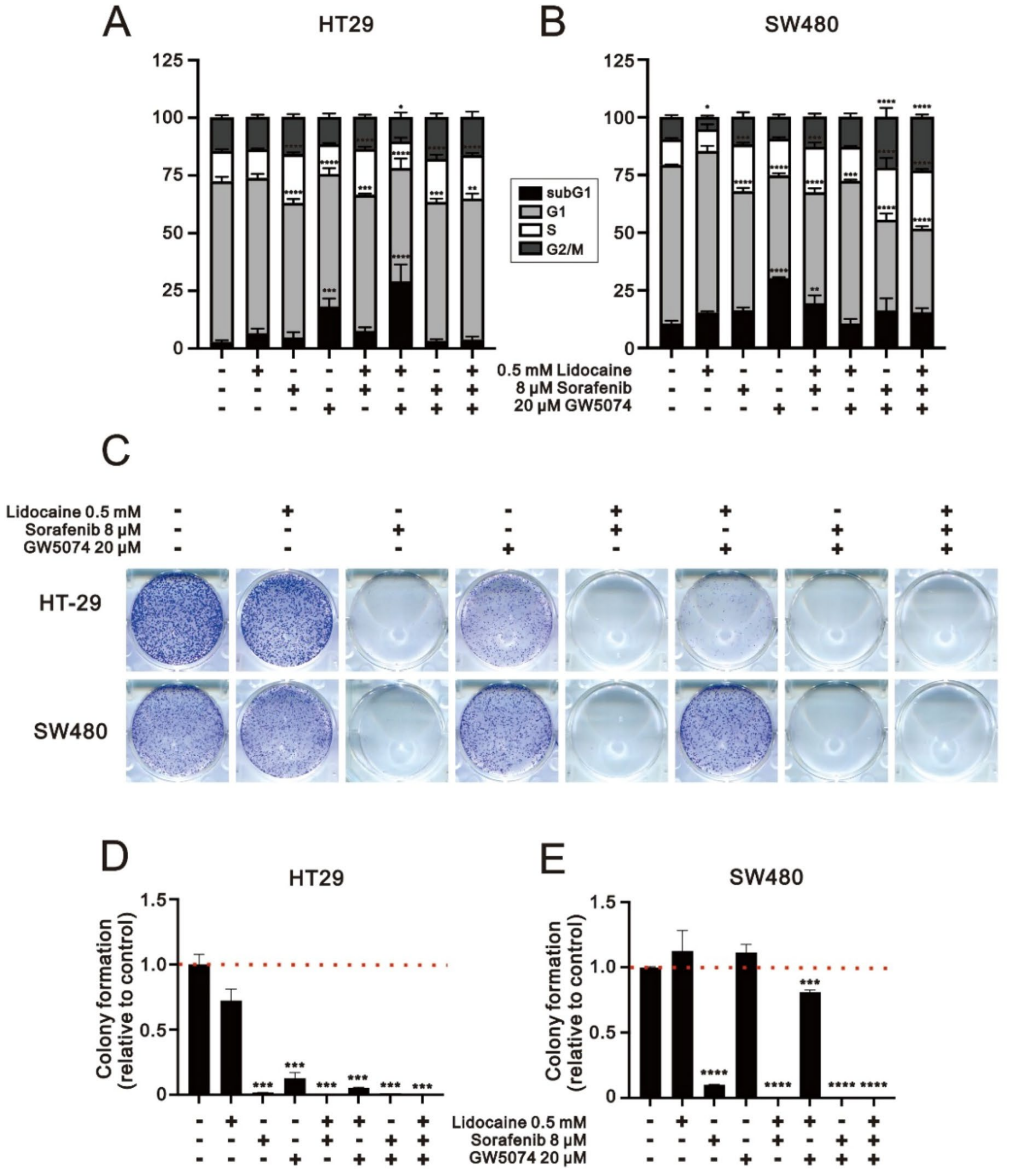
Effects of sorafenib, GW5074, and lidocaine on cell cycle distribution and clonogenic growth in CRC cells. (A) HT29 and (B) SW480 cells were treated for 48 h with 0.5 mM lidocaine, 8 μM sorafenib, or 20 μM GW5074, alone or in the indicated combinations. Cell cycle distribution was analysed by PI staining followed by flow cytometry. (C–E) HT29 (C, D) and SW480 (C, E) cells were treated for 14 days with 0.5 mM lidocaine, 8 μM sorafenib, or 20 μM GW5074, alone or in the indicated combinations. (C) Representative colony formation images are shown after methanol fixation and staining with 0.005% crystal violet. (D, E) Quantitative analysis of colony formation is presented as mean ± SEM from three biologically independent replicates. Statistical analysis was performed using two-way ANOVA followed by Dunnett’s multiple-comparisons test, with comparisons made against the vehicle control group. *p < 0.05, **p < 0.01, ***p < 0.001, ****p < 0.0001.

To evaluate the effects of lidocaine, sorafenib, GW5074, and their combinations on cell proliferation, we performed cell-cycle and colony formation assays. For convenience, lidocaine (L), sorafenib (S), and GW5074 (G) are referred to by their initials, and their combinations are denoted as LS, LG, SG, or LSG. In HT29 cells, GW5074 and LG significantly increased the subG1 population (Figure 2(A)). Sorafenib, GW5074, LS, LG, SG, and LSG significantly reduced the G1 population, whereas sorafenib, LS, SG, and LSG markedly increased the S-phase population. LG also significantly reduced the G2/M population. Similarly, in SW480 cells, GW5074 and LS significantly increased the subG1 population (Figure 2(B)). Sorafenib, GW5074, LS, LG, SG, and LSG significantly reduced the G1 population, whereas sorafenib, LS, SG, and LSG significantly increased the S-phase population. Lidocaine alone significantly reduced the G2/M population, whereas SG and LSG significantly increased it.

The colony formation assay assesses the long-term ability of adherent cells to survive and form clonal populations^23^. We used this assay to examine the effects of lidocaine, sorafenib, and GW5074 on the proliferative capacity of HT29 and SW480 cells (Figure 2(C)). Consistent with the cell-cycle findings, sorafenib, GW5074, LS, LG, SG, and LSG significantly inhibited colony formation in HT29 cells, indicating reduced proliferative capacity (*p* < 0.001, *p* < 0.001, *p* < 0.001, *p* < 0.001, *p* < 0.001, and *p* < 0.001, respectively, Figure 2(D)). In SW480 cells, sorafenib, LS, LG, SG, and LSG significantly reduced clonogenic potential, further supporting the growth-inhibitory effects of these treatments (*p* < 0.0001, *p* < 0.0001, *p* < 0.001, *p* < 0.0001, and *p* < 0.0001, respectively, Figure 2(E)).

### Effects of sorafenib, GW5074, and lidocaine on apoptosis and autophagy in CRC cells

**Figure 3.**
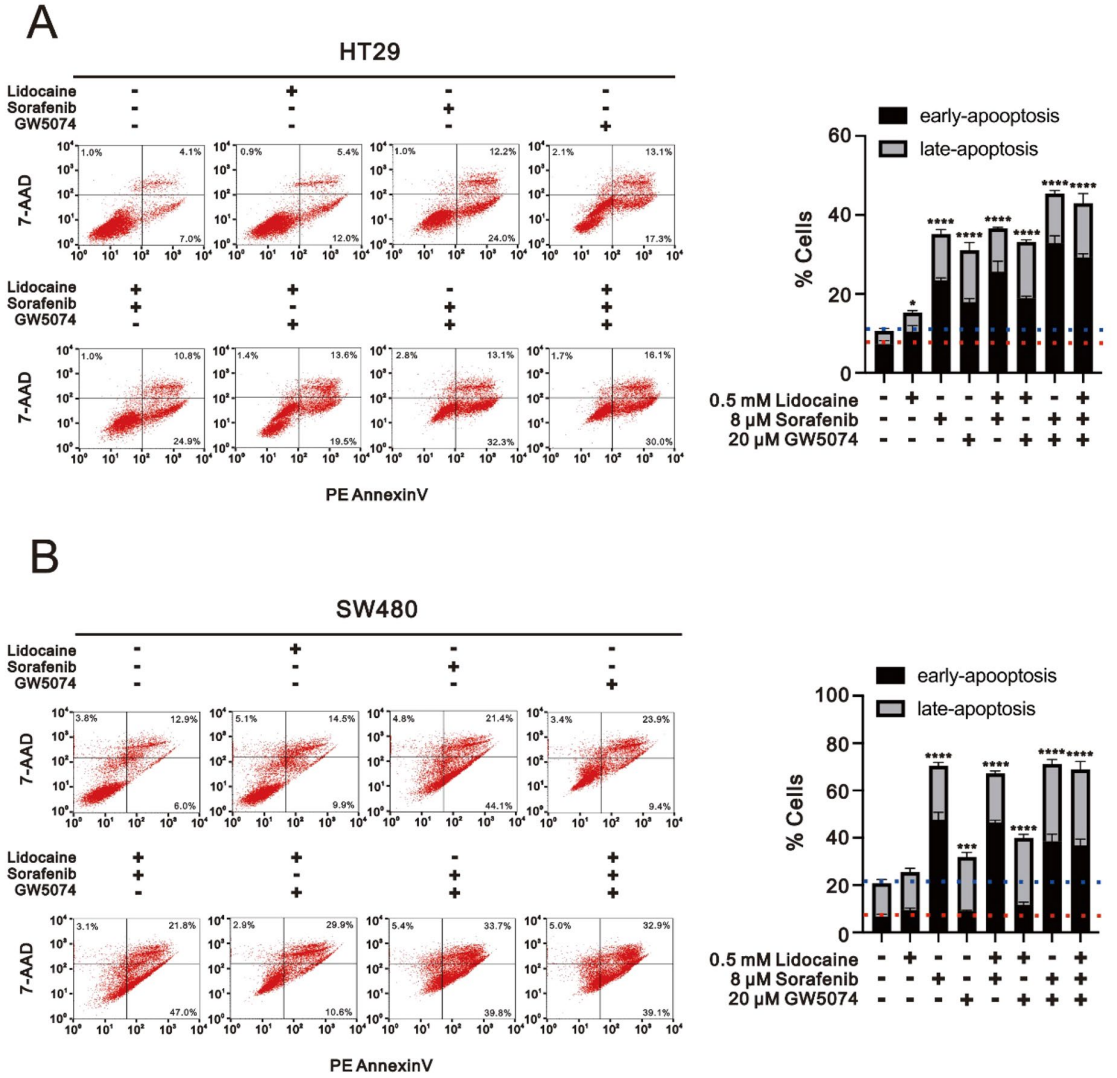
Effects of sorafenib, GW5074, and lidocaine on apoptosis in CRC cells. (A) HT29 and (B) SW480 cells were treated for 48 h with 0.5 mM lidocaine, 8 μM sorafenib, or 20 μM GW5074, alone or in the indicated combinations. Apoptosis was assessed by PE–Annexin V/7-AAD staining followed by flow cytometry. Bar graphs show the percentages of early and late apoptotic cells. Results are presented as mean ± SEM from three biologically independent replicates. Statistical analysis was performed using two-way ANOVA followed by Dunnett’s multiple-comparisons test, with comparisons made against the vehicle control group. *p < 0.05, ***p < 0.001, ****p < 0.0001.

Apoptosis and autophagy are distinct cellular processes involved in cell death and stress responses^24^. We next examined whether the observed cell-cycle perturbation and reduced proliferative capacity were associated with apoptosis. PE–Annexin V/7-AAD staining showed that lidocaine alone significantly induced apoptosis in HT29 cells, increasing both early and late apoptotic populations (Figure 3(A)). LS, LG, and LSG further increased apoptotic cell death. Sorafenib and GW5074 alone also significantly induced apoptosis, and their combination further increased the apoptotic population. In SW480 cells, lidocaine alone did not significantly induce early or late apoptosis (Figure 3(B)). However, sorafenib, GW5074, and their combinations with lidocaine substantially increased apoptosis. Sorafenib increased both early and late apoptotic populations, whereas GW5074 primarily increased late apoptosis. The effects of sorafenib on early and late apoptosis appeared to plateau, with no further increase observed after LS, SG, or LSG treatment.

**Figure 4.**
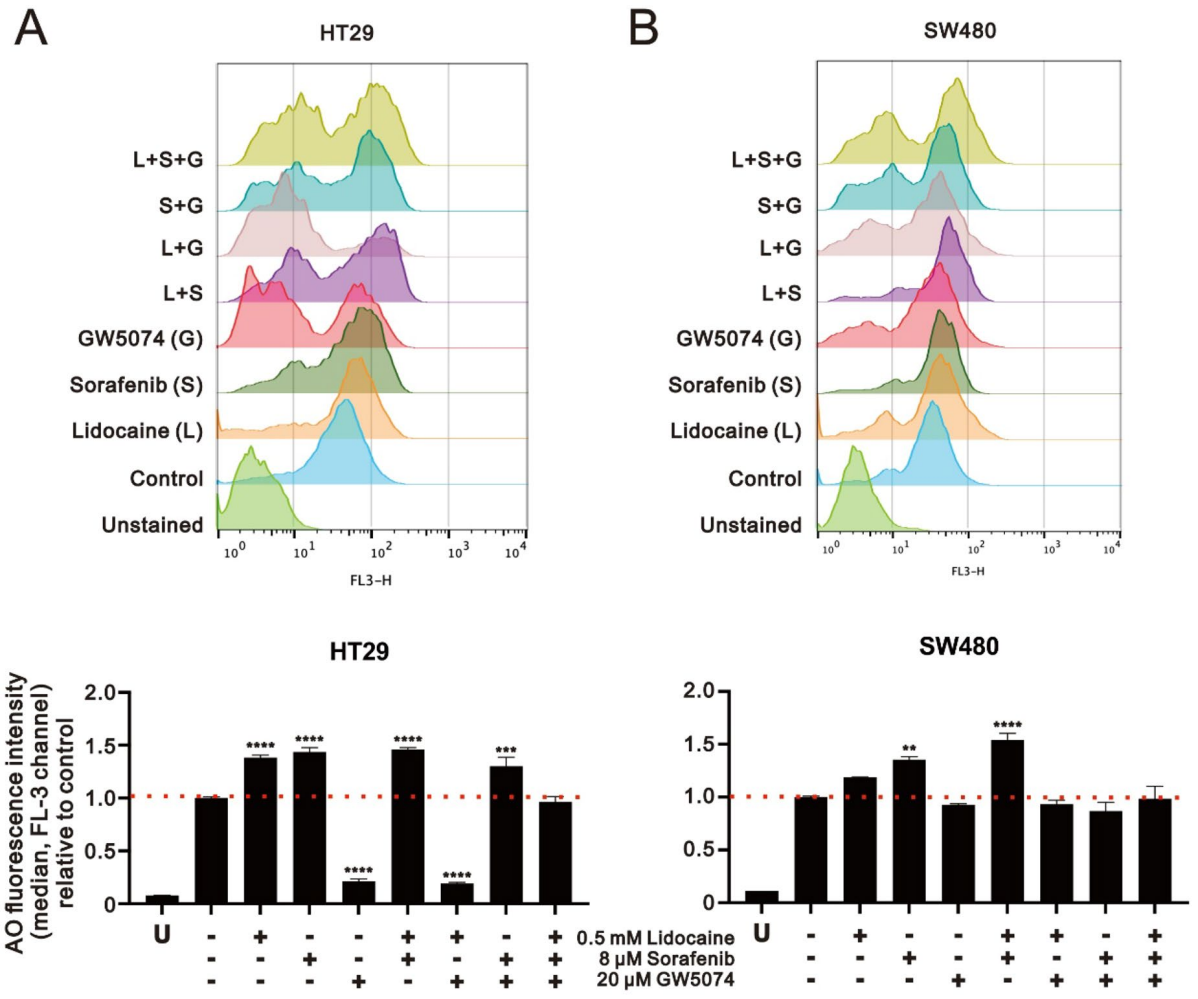
Effects of sorafenib, GW5074, and lidocaine on autophagy in CRC cells. (A) HT29 and (B) SW480 cells were treated for 48 h with 0.5 mM lidocaine, 8 μM sorafenib, or 20 μM GW5074, alone or in the indicated combinations. Cells were stained with acridine orange (AO), and autophagy was assessed and quantified by flow cytometry. Results are presented as mean ± SEM from three biologically independent replicates. Statistical analysis was performed using two-way ANOVA followed by Dunnett’s multiple-comparisons test, with comparisons made against the vehicle control group. **p < 0.01, ***p < 0.001, ****p < 0.0001.

We also assessed autophagic responses in HT29 and SW480 cells using AO staining. In HT29 cells, lidocaine alone significantly increased autophagic activity; similar induction was observed with LS, sorafenib alone, and SG (Figure 4(A)). These findings suggest stimulation of autophagy as part of the cellular stress response. In contrast, GW5074 alone and LG suppressed autophagic activity; SG and LSG also showed suppressive effects. In SW480 cells, sorafenib alone induced autophagy, and this effect was further enhanced by combination with lidocaine, suggesting increased autophagic activity after combined treatment (Figure 4(B)).

### Evaluation of ROS generation, lipid peroxidation, and hypoxia by sorafenib, GW5074, and lidocaine in CRC cells

**Figure 5.**
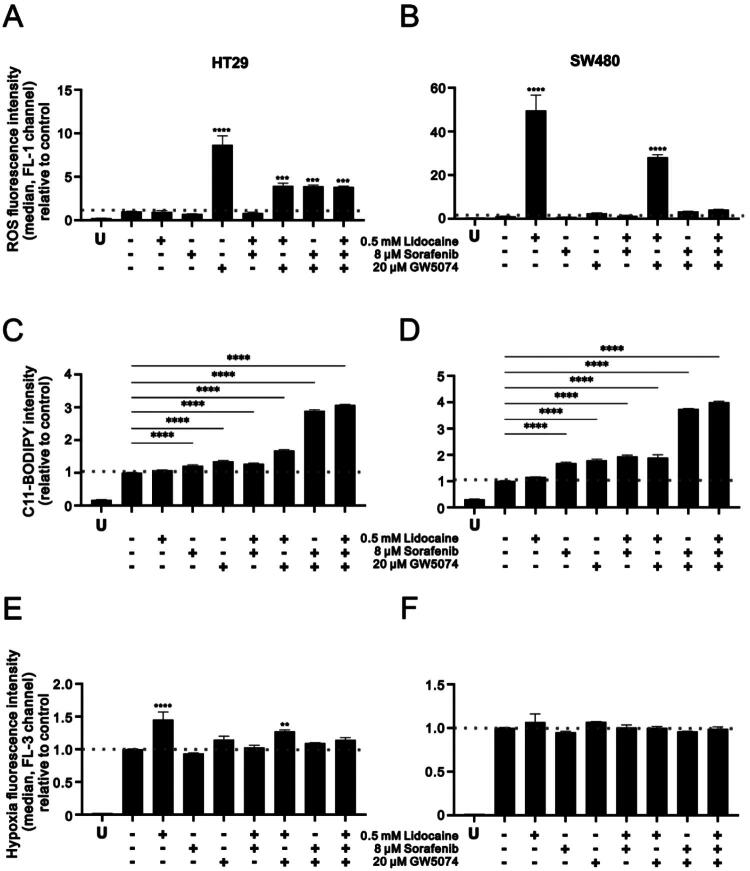
Effects of sorafenib, GW5074, and lidocaine on ROS levels, lipid peroxidation, and hypoxia in CRC cells. HT29 and SW480 cells were treated for 48 h with 0.5 mM lidocaine, 8 μM sorafenib, or 20 μM GW5074, alone or in the indicated combinations. (A, B) ROS levels were detected using DCFH-DA staining in HT29 and SW480 cells, respectively. (C, D) Lipid peroxidation was assessed using C11-BODIPY staining in HT29 and SW480 cells, respectively. (E, F) Hypoxia was evaluated using a nitroreductase activity dye and quantified by flow cytometry in HT29 and SW480 cells, respectively. Results are presented as mean ± SEM from three biologically independent replicates. Statistical analysis was performed using two-way ANOVA followed by Dunnett’s multiple-comparisons test, with comparisons made against the vehicle control group. **p < 0.01, ***p < 0.001, ****p < 0.0001.

Given the role of oxidative stress in apoptosis and autophagy^25^, we assessed intracellular ROS levels. In HT29 cells, GW5074 alone, LG, SG, and LSG significantly increased ROS production (*p* < 0.0001, *p* < 0.001, *p* < 0.001, and *p* < 0.001, respectively, Figure 5(A)). In contrast, lidocaine and sorafenib alone did not affect ROS levels, and GW5074 alone induced greater ROS production than any combination. In SW480 cells, lidocaine alone and LG markedly increased ROS levels (Figure 5(B)). However, sorafenib and GW5074 alone did not affect ROS production, and sorafenib suppressed lidocaine-induced ROS accumulation.

Because ROS accumulation can trigger ferroptosis^26^, we next evaluated lipid peroxidation using C11-BODIPY. Lidocaine alone did not significantly induce lipid peroxidation in either HT29 or SW480 cells (Figure 5(C,D)). However, LS and LG significantly increased lipid peroxidation, and LSG produced a robust increase. Sorafenib and GW5074 alone also promoted lipid peroxidation, whereas SG further enhanced this effect in both cell lines. These findings suggest increased ferroptotic vulnerability in CRC cells.

To assess tumour microenvironment-related stress, we evaluated cellular hypoxia using a fluorescent hypoxia probe activated by nitroreductase activity in hypoxic cells. In HT29 cells, lidocaine alone and LG significantly increased hypoxic signals, indicating enhanced cellular hypoxia (*p* < 0.0001 and *p* < 0.01, respectively, Figure 5(E)). In contrast, SW480 cells showed no significant hypoxic changes after any treatment (Figure 5(F)), suggesting context-dependent hypoxia-related responses.

### Evaluation of mitochondrial membrane potential by sorafenib, GW5074, and lidocaine in CRC cells

**Figure 6.**
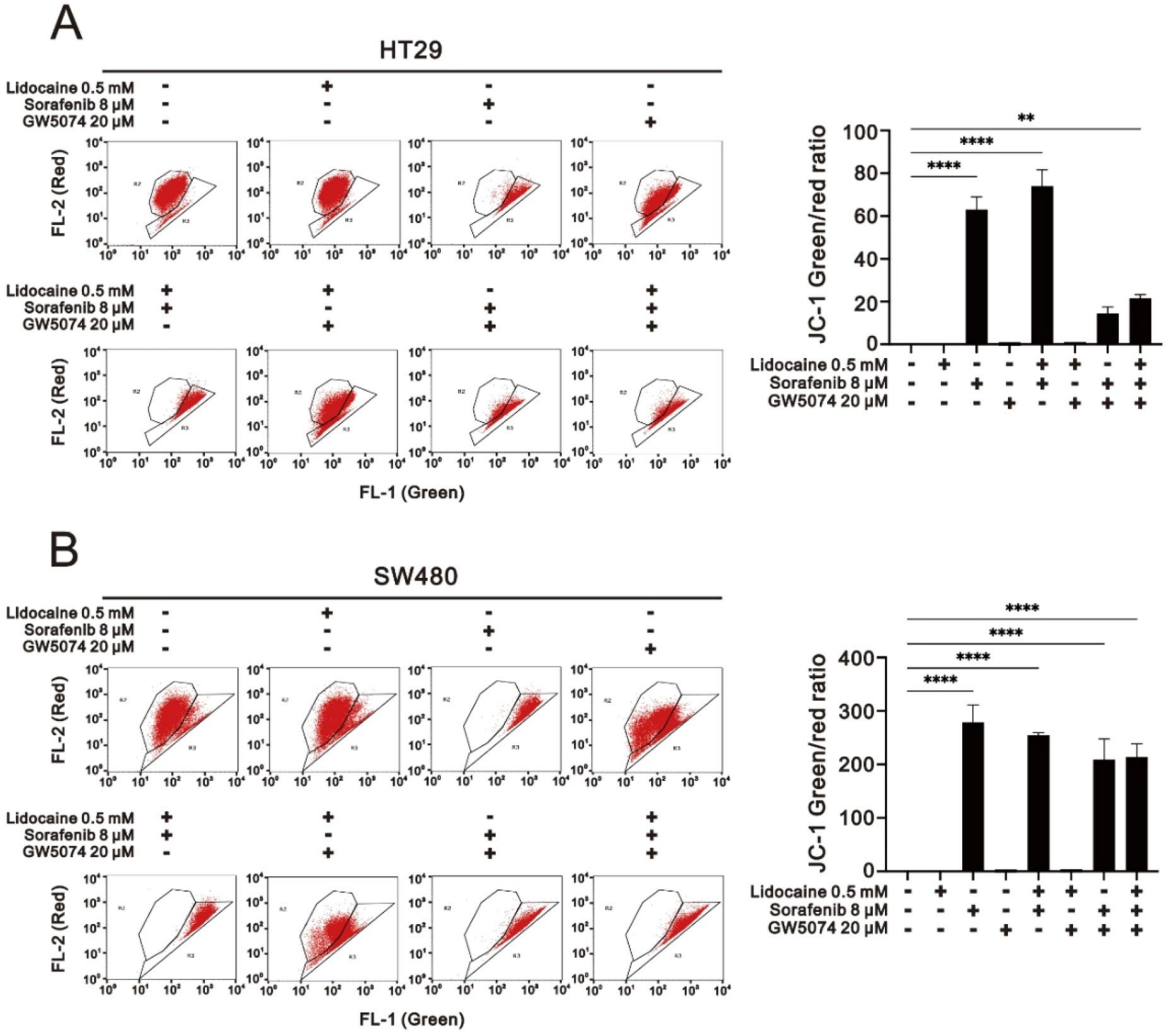
Effects of sorafenib, GW5074, and lidocaine on mitochondrial membrane potential in CRC cells. (A) HT29 and (B) SW480 cells were treated for 48 h with 0.5 mM lidocaine, 8 μM sorafenib, or 20 μM GW5074, alone or in the indicated combinations. Mitochondrial membrane potential was assessed using JC-1 staining and quantified by flow cytometry. Results are presented as mean ± SEM from three biologically independent replicates. Statistical analysis was performed using two-way ANOVA followed by Dunnett’s multiple-comparisons test, with comparisons made against the vehicle control group. **p < 0.01, ****p < 0.0001.

To determine whether oxidative stress and lipid peroxidation were associated with mitochondrial dysfunction, we assessed mitochondrial membrane potential (ΔΨm) using the JC-1 assay. JC-1 staining distinguishes red-fluorescent aggregates in healthy mitochondria from green-fluorescent monomers in depolarised mitochondria. In HT29 cells, neither lidocaine nor GW5074 alone induced significant mitochondrial depolarisation (Figure 6(A)). However, sorafenib alone markedly reduced ΔΨm, and this effect was further enhanced by combination with lidocaine. Conversely, SG and LSG attenuated sorafenib-induced ΔΨm loss. In SW480 cells, lidocaine and GW5074 alone similarly did not induce significant mitochondrial depolarisation (Figure 6(B)). Sorafenib alone markedly reduced ΔΨm, whereas LS, SG, and LSG suppressed this effect. These findings suggest that sorafenib-induced ΔΨm loss may be associated with lipid peroxidation-related signalling rather than ROS accumulation. The additive effect of LS was observed in HT-29 cells but not in SW480 cells, whereas the suppressive effects of SG and LSG were observed in both cell lines.

### Evaluation of synergistic effects between amide-type local anaesthetics and sorafenib or GW5074 in CRC cells

**Figure 7.**
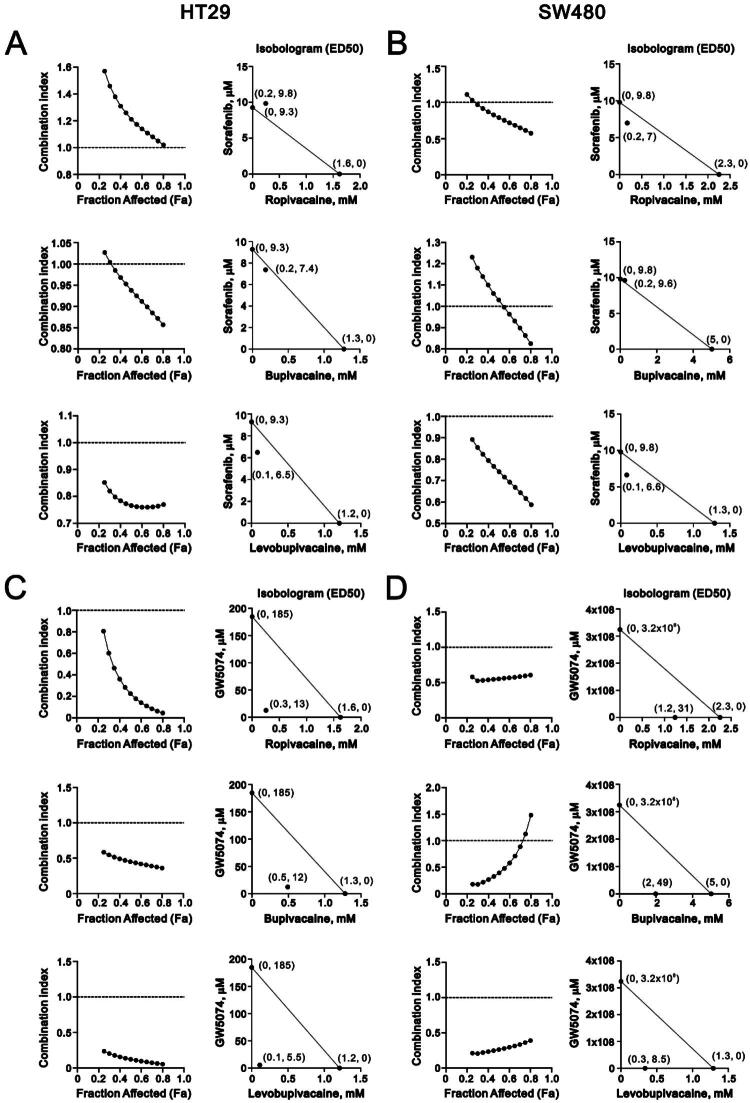
Drug interactions between amide-type local anaesthetics and Raf-targeted agents in CRC cells using combination index (CI) analysis. (A) HT29 and (B) SW480 cells were treated for 48 h with increasing concentrations of sorafenib (0, 0.625, 1.25, 2.5, 5, 10, 20, 40, 80, or 160 μM) in combination with ropivacaine (0, 0.06, 0.125, 0.25, 0.5, 1, 2, or 4 μM), bupivacaine (0, 0.06, 0.125, 0.25, 0.5, 1, 2, or 4 μM), or levobupivacaine (0, 0.03, 0.06, 0.12, 0.25, 0.5, 1, or 2 μM). (C) HT29 and (D) SW480 cells were treated for 48 h with increasing concentrations of GW5074 (0, 0.39, 0.78, 1.56, 3.125, 6.25, 12.5, 25, 50, or 100 μM) in combination with the same amide-type local anaesthetics at the indicated concentrations. Cell viability was assessed using the MTT assay. ED50 isobolograms and CI plots were generated using CalcuSyn software.

To evaluate whether the observed synergistic interactions are unique to lidocaine or extend to other amide-type local anaesthetics, we tested ropivacaine, bupivacaine, and levobupivacaine in combination with sorafenib or GW5074 in CRC cells. Similar to lidocaine, ropivacaine in combination with sorafenib exhibited an antagonistic effect in HT29 cells (Figure 7(A)), while both bupivacaine and levobupivacaine demonstrated synergistic interactions. In SW480 cells, all three local anaesthetics: ropivacaine, bupivacaine, and levobupivacaine showed synergistic effects when combined with sorafenib (Figure 7(B)). Ropivacaine, bupivacaine, and levobupivacaine combined with GW5074 exhibited synergistic interactions in both HT29 and SW480 cells (Figure 7(C,D)). Specifically, ropivacaine, bupivacaine, and levobupivacaine increased GW5074 responsiveness by 14- and 1.03 × 10^7^-fold, 15.1- and 6.5 × 10^6^-fold, and 33.6- and 3.8 × 10^7^-fold in HT29 and SW480 cells, respectively. Overall, levobupivacaine showed the strongest enhancement of synergy with sorafenib or GW5074 in both cell lines.

## Discussion

Sorafenib, a multikinase inhibitor, and GW5074, a selective c-Raf inhibitor, are promising targeted therapies for CRC, but their efficacy is limited by therapeutic resistance. This study systematically examined the combined effects of lidocaine, sorafenib, and GW5074 in HT29 and SW480 CRC cells. To determine whether lidocaine enhances the effects of these kinase inhibitors, we focused on key cellular processes, including cell-cycle progression, lipid peroxidation, mitochondrial dysfunction, and hypoxia-related signalling. Our findings show that these combinations modulate cell-cycle progression, activate cell death-related pathways, and amplify cellular stress responses. CI analysis demonstrated that lidocaine, ropivacaine, bupivacaine, and levobupivacaine combined with GW5074 showed synergistic interactions in both HT29 and SW480 cells. However, lidocaine and ropivacaine did not synergize with sorafenib in HT29 cells. Collectively, these findings suggest that local anaesthetics may enhance the effects of selected kinase-targeted therapies in a cell context-dependent manner.

HT29 and SW480 cells differ in phenotype and molecular background. HT29 cells have been reported to display a more epithelial phenotype with low migratory capacity, whereas SW480 cells show a mesenchymal phenotype with higher migratory activity^27^. In our study, SG consistently produced strong synergistic effects in both cell lines, whereas the effects of lidocaine were context dependent. In HT29 cells, LS showed antagonism, whereas LG retained synergistic activity. In contrast, lidocaine enhanced synergistic interactions with both sorafenib and GW5074 in SW480 cells. These differences may reflect the distinct molecular features of each cell line. CRC progression is commonly associated with inactivation of tumour suppressor genes such as APC, PTEN, and TP53 and activation of oncogenes such as RAS, BRAF, and PIK3CA^28^. HT29 cells harbour BRAF mutations and partially functional p53 and rely substantially on MAPK-driven differentiation signalling,^28^ which may limit the ability of lidocaine to enhance sorafenib activity. Conversely, SW480 cells harbour KRAS and APC mutations and complete loss of p53, with altered redox balance and metabolic reprogramming^29^, which may increase susceptibility to the pro-oxidant and stress-enhancing effects of lidocaine. Thus, the different synergy patterns observed between HT29 and SW480 cells may reflect intrinsic differences in oncogenic drivers, redox status, and stress-response mechanisms. Similar context-dependent differences in drug responsiveness have been reported previously^30^^,^^31^, including differential sensitivity to 5-FU among CRC cell lines, with SW480 displaying higher sensitivity than HT29^32^.

The interplay between cell-cycle progression and cell death pathway activation is an important determinant of therapeutic response^1^^,^^33^. Our findings suggest that this relationship varies according to the drug combination and cellular context. In SW480 cells, SG and LSG induced G2/M accumulation. Because the G2/M phase is often considered the most radiosensitive cell-cycle stage, this finding suggests that these combinations may have radiosensitizing potential, particularly in SW480 cells^34^^,^^35^. This shift may partly explain the stronger synergistic responses observed in SW480 cells than in HT29 cells. In both cell lines, treatments generally reduced the G1 population and increased the S-phase population, suggesting interference with the G1/S transition. This may involve modulation of cell-cycle regulators, such as cyclin D1/CDK4/6, or activation of intra-S-phase DNA damage checkpoints^36^. S-phase accumulation is consistent with replication stress and impaired cell-cycle progression. Together with subG1 accumulation, these findings suggest that replication stress and checkpoint activation may contribute to apoptosis induction^37^. The differential effects on G2/M progression further indicate that the regulatory mechanisms underlying these responses differ between the two cell lines.

Autophagy is commonly recognised as a stress-induced pro-survival mechanism, although excessive autophagic vacuole accumulation may disrupt cellular function and contribute to non-apoptotic cell death^38^. Therefore, the role of autophagy in therapeutic response is highly context dependent. In HT29 cells, lidocaine and sorafenib-induced autophagy, whereas GW5074 suppressed it. This divergence suggests that autophagy in HT29 cells may initially function as a protective response to mild drug-induced stress but may contribute to cell death when stress becomes more severe, particularly after sorafenib treatment. Sorafenib-induced autophagy has been linked to stress-response pathways, including mTOR modulation.^37^ In SW480 cells, lidocaine enhanced sorafenib-induced autophagy, suggesting that these agents may converge on overlapping stress-response pathways, potentially through increased mTOR suppression or intensified cellular damage. These findings highlight the complexity of autophagic regulation and indicate that autophagy may either promote survival or facilitate cell death depending on tumour genotype and treatment context.

Sorafenib inhibits several tyrosine kinase receptors and exerts antitumor effects through mechanisms that include suppression of proliferation and induction of apoptosis, depending on cancer type^39–42^. Improving sorafenib efficacy requires a clearer understanding of the molecular, cellular, and microenvironmental mechanisms underlying response and resistance^39^^,^^40^. Previous work showed that sorafenib combined with GW5074 exerted synergistic anticancer effects through translocation of pC-Raf^S338^ and pDAPK^S308^ from mitochondria to the cytoplasm, along with decreased mitochondrial membrane potential and increased ROS generation^9^. Our previous study demonstrated that the synergistic effects of GW5074 and sorafenib were mainly found in mitochondrial functions, including ROS generation, membrane potential disruption, and fission–fusion dynamics in HCT116 and LoVo CRC cells^8^. Modified GW5074 structures have also been evaluated in a phase I targeted combination trial of sorafenib and GW5074 in refractory advanced solid cancers^43^. However, GW5074 has limited solubility, which may restrict its therapeutic development. In this study, we used a drug-repurposing approach to evaluate lidocaine and other local anaesthetics as potential sensitisers for sorafenib, GW5074, or their combination in CRC cells. All tested local anaesthetics showed synergy with GW5074, but not consistently with sorafenib, in HT29 and SW480 cells. Notably, the highest lidocaine concentration used in our *in vitro* experiments was 4 mM, equivalent to 0.108%, which is lower than the concentrations typically used for local anaesthesia in plastic surgery and dermatology (1%) or dental practice (2%). These findings suggest that lidocaine and other local anaesthetics may help reduce the effective concentration of GW5074 and support further investigation of GW5074-based combinations. According to the consensus molecular subtype (CMS) classification of CRC cell lines^44^, HCT116 and LoVo cells are CMS1, HT29 is CMS2, and SW480 is CMS4. The different cytotoxic mechanisms of sorafenib and GW5074 across CRC cell lines suggest that the potential application of lidocaine- and sorafenib-based combinations may depend on the cellular context of the cancer cells. These differences also highlight the importance of selecting targeted therapies according to tumour subtype and molecular background.

## Data Availability

The datasets generated and/or analysed during this study are available from the corresponding author upon reasonable request.
